# Impact of the change in WHO’s severe pneumonia case definition on hospitalized pneumonia epidemiology: case studies from six countries

**DOI:** 10.2471/BLT.18.223271

**Published:** 2019-03-27

**Authors:** Fiona M Russell, Rita Reyburn, Jocelyn Chan, Evelyn Tuivaga, Ruth Lim, Jana Lai, Hoang Minh Tu Van, Molina Choummanivong, Vanphanom Sychareun, Dung Khu Thi Khanh, Margaret de Campo, Penny Enarson, Stephen Graham, Sophie La Vincente, Tuya Mungan, Claire von Mollendorf, Grant Mackenzie, Kim Mulholland

**Affiliations:** a Centre for International Child Health, Department of Paediatrics, University of Melbourne, 50 Flemington Road, Parkville, Melbourne, 3052, Australia.; b New Vaccines, Murdoch Children’s Research Institute, Melbourne, Australia.; c Paediatrics Department, Ministry of Health and Medical Services, Suva, Fiji.; d Paediatric Department, Children’s Hospital No. 2, Ho Chi Minh City, Viet Nam.; e Faculty of Postgraduate Studies, University of Health Sciences, Vientiane, Lao People’s Democratic Republic.; f Neonatal Department, National Children’s Hospital, Hanoi, Viet Nam.; g Department of Radiology, The University of Melbourne, Melbourne, Australia.; h International Union Against Tuberculosis and Lung Disease, Paris, France.

## Abstract

**Objective:**

To quantify the impact of the change in definition of severe pneumonia on documented pneumonia burden.

**Methods:**

We reviewed existing data acquired during observational hospitalized pneumonia studies, before the introduction of the pneumococcal conjugate vaccine, in infants aged 2–23 months from Fiji, Gambia, Lao People's Democratic Republic, Malawi, Mongolia and Viet Nam. We used clinical data to calculate the percentage of all-cause pneumonia hospitalizations with severe pneumonia, and with primary end-point consolidation, according to both the 2005 or 2013 World Health Organization (WHO) definitions. Where population data were available, we also calculated the incidence of severe pneumonia hospitalizations according to the different definitions.

**Findings:**

At six of the seven sites, the percentages of all-cause pneumonia hospitalizations due to severe pneumonia were significantly less (*P* < 0.001) according to the 2013 WHO definition compared with the 2005 definition. However, the percentage of severe pneumonia hospitalizations, according to the two definitions of severe pneumonia, with primary end-point consolidation varied little within each site. The annual incidences of severe pneumonia hospitalizations per 100 000 infants were significantly less (all *P* < 0.001) according to the 2013 definition compared with the 2005 definition, ranging from a difference of −301.0 (95% confidence interval, CI: −405.2 to −196.8) in Fiji to −3242.6 (95% CI: −3695.2 to −2789.9) in the Gambia.

**Conclusion:**

The revision of WHO’s definition of severe pneumonia affects pneumonia epidemiology, and hence the interpretation of any pneumonia intervention impact evaluation.

## Résumé

**Objectif:**

Quantifier l'impact de la modification de la définition de pneumonie sévère sur le fardeau documenté de la pneumonie.

**Méthodes:**

Nous avons examiné les données existantes obtenues dans le cadre d'études d'observation de la pneumonie hospitalière, avant l'introduction du vaccin antipneumococcique conjugué, chez des nourrissons âgés de 2 à 23 mois aux Fidji, en Gambie, au Malawi, en Mongolie, en République démocratique populaire lao et au Viet Nam. Nous avons utilisé des données cliniques pour calculer le pourcentage d'hospitalisations pour une pneumonie toutes causes confondues avec une pneumonie sévère, et avec une consolidation des critères d'évaluation primaire, selon les définitions de l'Organisation mondiale de la Santé (OMS) de 2005 et 2013. Lorsque des données démographiques étaient disponibles, nous avons également calculé l'incidence des hospitalisations pour une pneumonie sévère selon les différentes définitions.

**Résultats:**

Dans six des sept sites, les pourcentages d'hospitalisations pour une pneumonie toutes causes confondues en raison d'une pneumonie sévère étaient nettement plus faibles (*P* < 0,001) selon la définition de l'OMS de 2013 en comparaison avec la définition de 2005. Néanmoins, le pourcentage d'hospitalisations pour une pneumonie sévère, selon les deux définitions de la pneumonie sévère, avec une consolidation des critères d'évaluation primaire variait peu dans chaque site. Les incidences annuelles d'hospitalisations pour une pneumonie sévère pour 100 000 nourrissons étaient nettement plus faibles (total *P* < 0,001) selon la définition de 2013 en comparaison avec la définition de 2005, la différence allant de −301,0 (intervalle de confiance, IC, à 95%: de −405,2 à −196,8) aux Fidji à −3242,6 (IC à 95%I: −de 3695,2 à −2789,9) en Gambie.

**Conclusion:**

La révision de la définition de l'OMS de la pneumonie sévère affecte l'épidémiologie de la pneumonie et, par conséquent, l'interprétation de toute évaluation de l'impact d'une intervention contre la pneumonie.****

## Resumen

**Objetivo:**

Cuantificar el impacto del cambio en la definición de neumonía grave sobre la carga de neumonía documentada.

**Métodos:**

Se revisaron los datos existentes adquiridos durante los estudios observacionales de neumonía hospitalizada, antes de la introducción de la vacuna antineumocócica conjugada, en lactantes de 2 a 23 meses de edad procedentes de Fiji, Gambia, Malawi, Mongolia, la República Democrática Popular Lao y Vietnam. Se utilizaron datos clínicos para calcular el porcentaje de hospitalizaciones por neumonía de cualquier causa con neumonía grave, y con consolidación del punto final primario, de acuerdo con las definiciones de la Organización Mundial de la Salud (OMS) de 2005 o 2013. Cuando se dispuso de datos demográficos, también se calculó la incidencia de las hospitalizaciones por neumonía grave de acuerdo con las diferentes definiciones.

**Resultados:**

En seis de los siete sitios, los porcentajes de hospitalizaciones por neumonía de cualquier causa con neumonía grave fueron significativamente menores (p < 0,001) según la definición de la OMS de 2013 en comparación con la de 2005. Sin embargo, el porcentaje de hospitalizaciones por neumonía grave, según las dos definiciones de neumonía grave, con consolidación del punto final primario varió poco dentro de cada sitio. La incidencia anual de hospitalizaciones por neumonía grave por cada 100.000 lactantes fue significativamente menor (total p < 0,001) según la definición de 2013 en comparación con la de 2005 y osciló entre una diferencia de −301,0 (intervalo de confianza, IC, del 95 %: −405,2 a −196,8) en Fiji y −3242,6 (IC del 95 %: −3695,2 a −2789,9) en Gambia.

**Conclusión:**

La revisión de la definición de neumonía grave de la OMS afecta a la epidemiología de la neumonía y, por tanto, a la interpretación de cualquier evaluación del impacto de una intervención para la neumonía.

## ملخص

الغرض قياس تأثير التغيير في تعريف الالتهاب الرئوي الشديد على عبء الالتهاب الرئوي الموثق.

الطريقة لقد قمنا باستعراض البيانات التي تم الحصول عليها أثناء دراسات الالتهاب الرئوي في المستشفيات تحت الملاحظة، قبل طرح لقاح المكورات الرئوية، في الرضع الذين تتراوح أعمارهم بين 2 و3 أشهر من فيجي، وغامبيا، وجمهورية لاو الديمقراطية الشعبية، وملاوي، ومنغوليا، وفييت نام. واستخدمنا البيانات السريرية لحساب النسبة المئوية لعلاج حالات الإصابة بالالتهاب الرئوي الشديد بالمستشفيات مع كل الأسباب، ومع توحيد نقطة النهاية الأولية، وفقا لكل من تعريفات منظمة الصحة العالمية (WHO) عامي 2005 أو 2013. وحيثما كانت البيانات السكانية متاحة، قمنا أيضاً بحساب معدل العلاج بالمستشفى لحالات الالتهاب الرئوي الشديد وفقاً للتعريفات المختلفة.

**النتائج:**

في ستة من المواقع السبعة، كانت النسب المئوية لحالات العلاج بالمستشفيات نتيجة الإصابة بالالتهاب الرئوي الشديد بالمستشفيات مع كل الأسباب أقل بشكل ملموس (نسبة الاحتمال < 0.001) وفقًا لتعريف منظمة الصحة العالمية لعام 2013 مقارنةً بتعريف عام 2005. إلا أن النسبة المئوية لحالات علاج الالتهاب الرئوي الشديد بالمستشفيات، وفقا لتعريفي الالتهاب الرئوي الشديد، مع توحيد نقطة النهاية الأولية، تباينت قليلا داخل كل موقع. كان المعدل السنوي لحالات العلاج بالمستشفيات نتيجة الإصابة بالالتهاب الرئوي الشديد من كل الأسباب أقل بشكل ملموس (نسبة الاحتمال < 0.001) وفقًا لتعريف عام 2013 مقارنةً بتعريف عام 2005، يتراوح من اختلاف قدره -301.0 (فاصل الثقة 95%: 405.2- إلى 196.8-) في فيجي إلى 3242.6- (فاصل الثقة 95‏%: 3695.2- إلى 2789.9-) في غامبيا.

**الاستنتاج:**

إن مراجعة تعريف منظمة الصحة العالمية (WHO) للالتهاب الرئوي الشديد يؤثر على الانتشار الوبائي الالتهاب الرئوي، ومن ثم يؤثر على تحليل أي تقييم لتأثير التدخل العلاجي للالتهاب الرئوي.

## 摘要

**目的:**

旨在量化重症肺炎定义的变化对已记录肺炎负担的影响。

**方法:**

我们回顾了斐济、冈比亚、老挝人民民主共和国、马拉维、蒙古和越南六国在引入肺炎球菌结合疫苗之前，2–23 个月大的婴儿在住院肺炎的观察性研究中获得的现有数据。根据 2005 年和 2013 年世界卫生组织的定义，我们使用临床数据计算了重症肺炎和主要终点巩固的全因肺炎住院患者的百分比。基于可获得的人口数据，我们还根据不同定义计算了重症肺炎住院治疗的发病率。

**结果:**

根据 2013 年与 2005 年世界卫生组织的定义对比结果，发现 6 个地点（共 7 个）中因重症肺炎而住院的全因肺炎患者百分比显著下降（*P* < 0.001）。然而，根据重症肺炎的两类定义，重症肺炎与主要终点巩固住院患者的百分比在每个地点的差异并不显著。根据 2013 年与 2005 年的定义对比结果，每 100，000 名婴儿中因重症肺炎住院的年发病率显著下降（均为 *P* < 0.001），差异范围从斐济的 301.0（95% 置信区间，CI： 405.2 至 196.8）到冈比亚的 3242.6（95% CI： 3695.2 至  2789.9）。

**结论:**

世卫组织对重症肺炎定义的修订会影响肺炎流行病学，从而对任何肺炎干预评估的解释造成影响。****

## Резюме

**Цель:**

Количественная оценка воздействия изменений в определении тяжелой формы пневмонии на документированное бремя заболевания.

**Методы:**

Авторы изучили имеющиеся данные, собранные во время обсервационных исследований пневмонии у госпитализированных больных до начала использования пневмококковой конъюгированной вакцины у детей в возрасте от 2 до 23 месяцев из Вьетнама, Гамбии, Лаосской Народно-Демократической Республики, Малави, Монголии и Фиджи. Клинические данные использовались для расчета процентной доли госпитализации пациентов с тяжелой формой пневмонии, независимо от ее причины, с консолидацией по основной конечной точке в соответствии с двумя разными определениями ВОЗ: от 2005 года и от 2013 года. При наличии популяционных данных авторы также рассчитывали частоту госпитализации пациентов с тяжелой формой пневмонии в соответствии с этими двумя определениями.

**Результаты:**

В шести из семи исследованных стран доля госпитализации пациентов с тяжелой формой пневмонии, независимо от причины заболевания, была значительно ниже (*P* < 0,001) при использовании определения ВОЗ от 2013 года, чем при использовании определения от 2005 года. Однако процентная доля госпитализации с тяжелой формой пневмонии, согласно двум разным определениям такой тяжелой формы, с консолидацией по основной конечной точке мало различалась в разных странах. Ежегодная частота случаев госпитализации с тяжелой формой пневмонии на 100 000 младенцев была значительно ниже (все значения *P* < 0,001) при использовании определения от 2013 года по сравнению с определением от 2005 года. Разница составляла от −301,0 случая (95%-й ДИ: −от 405,2 до −196,8) для Фиджи до −3242,6 случая (95%-й ДИ: −от 3695,2 до −2789,9) для Гамбии.

**Вывод:**

Пересмотр определений ВОЗ для тяжелой формы пневмонии влияет на эпидемиологию заболевания и, следовательно, на интерпретацию оценки воздействия любого вмешательства, связанного с этим заболеванием.****

## Introduction

Pneumonia is the leading cause of post-neonatal deaths in children younger than 5 years, with many of the deaths occurring in low- and middle-income countries.^1^^,^^2^ Several interventions exist that prevent pneumonia,^3^ including the pneumococcal conjugate vaccine; however, this vaccine is relatively expensive, meaning that governments require evidence of its health benefits in routine use. Such evidence is often provided by hospital admission data, but this requires a standardized pneumonia case definition as well as uniform admission criteria for pneumonia. To improve the case management of pneumonia in low- and middle-income countries, the World Health Organization (WHO) developed the Integrated Management of Childhood Illness guidelines in 1995.

In 2005, these guidelines^4^ defined severe pneumonia as the presence of cough or difficulty in breathing and tachypnoea (> 50 breaths per minute for children aged 2–11 months and > 40 breaths per minute for children aged 12–59 months) plus lower chest indrawing, and one or more of the general danger signs, which include: an inability to drink; persistent vomiting; convulsions; lethargy; unconsciousness; stridor in a calm child; severe malnutrition (as documented in the medical records); central cyanosis; or a saturation of oxygen of < 90% in room air. Many low- and middle-income countries adopted these criteria. However, equivalence and non-inferiority studies found no difference in treatment failure rates between patients with pneumonia with lower chest indrawing who were treated with parenteral antibiotics in hospital, and outpatient treatment with oral antibiotics.^5^^,^^6^ As a result, WHO modified the classification of pneumonia severity;^7^ the 2013 definition of severe pneumonia requiring hospital admission is the presence of cough or difficulty in breathing and tachypnoea, plus one or more of the general danger signs, but not lower chest indrawing. 

Between 2010 and 2013, before WHO’s case definition for severe pneumonia was revised, more than 54 countries, supported by Gavi, the Vaccine Alliance, implemented the pneumococcal conjugate vaccine.^8^ The impact of this change in case definition on the documented burden of severe pneumonia is unclear.

We therefore assess clinical data describing pneumonia hospitalizations in six countries and determine the effect of the revised 2013 WHO definition of severe pneumonia on the proportion of all-cause hospitalized pneumonia cases that are classified as severe, and on the incidence of severe hospitalized pneumonia. We also explore the impact of this change in the case definition on radiological pneumonia, that is, patients with primary end-point consolidation evident from chest X-ray. Using observational data from seven sites, two from the African Region and five from the Western Pacific Region, we describe the epidemiology of severe pneumonia using both the 2005 and 2013 WHO definitions. We quantify the apparent reduction of hospitalized pneumonia cases with severe pneumonia, as well as the apparent fall in annual incidence of severe pneumonia hospitalizations with primary end-point consolidation, resulting directly from the change in definition of severe pneumonia.

## Methods

### Data sources

We requested data from investigators undertaking retrospective or prospective hospitalized pneumonia studies in children aged 2–23 months from seven sites in six countries before the introduction of the pneumococcal conjugate vaccine: Fiji, Gambia,^9^ Lao People's Democratic Republic, Malawi,^10^^–^^12^ Mongolia and Viet Nam (with separate data sets for Hanoi and Ho Chi Minh City; Table 1). We reviewed the individual medical records of patients from all sites and extracted clinical information, including: age, respiratory rate and the presence of lower chest wall indrawing, stridor when calm or any general danger signs (inability to drink or vomiting everything, convulsions, lethargy or unconsciousness). Further details on the data collection methods at each study site are available from the corresponding author.

**Table 1:** Impact study of WHO’s severe pneumonia case definition change: data sources in six countries

Country, city or region	Hospital (level of care)	Study design	Period	No. all-cause pneumonia hospitalizations of children aged 2–23 months	WHO guidelines used
Fiji, Suva	Colonial War Memorial Hospital (tertiary)	Retrospective	2007 to 2011	3254	2005
Gambia, Upper River Region	Basse health centre (primary and secondary)	Prospective^9^	May 2008 to Aug 2009	953	None; study-specific criteria^13^
Lao People's Democratic Republic, Vientiane	Mahosot, Settathirath, Hospital 103, National Child, Mother and Child Hospitals (tertiary); nine district hospitals (secondary)	Retrospective	2011 to 2013	678	2005
Malawi	16 out of 24 district hospitals (secondary)	Prospective^10^^–^^12^	Oct 2000 to Jun 2003	15 709	2005^a^
Mongolia, Ulaanbaatar	Four district hospitals (secondary), National Mother and Child Hospital (tertiary)	Prospective	April 2015 to May 2016	3051^b^	2005
Viet Nam, Hanoi	National Children’s Hospital (tertiary)	Retrospective	2010	262^c^	None
Viet Nam, Ho Chi Minh City	Children’s Hospital No. 2 (tertiary)	Retrospective	2010	380	2005

WHO: World Health Organization.

^a^ Guidelines used were the same as the 2005 definition, although the study was performed before these guidelines were available.

^b^ Only children with clinical information included.

^c^ Every 10th pneumonia discharge in 2010 was included.

### Data processing

We used the extracted clinical information to classify pneumonia cases as severe according to the 2005 and 2013 WHO definitions of severe pneumonia and calculated the median age (in months) and interquartile range of patients for each site. We compared the different median ages of the children with severe pneumonia according to the different definitions using the non-parametric *K*-sample test on the equality of medians. We also calculated the percentage of all-cause pneumonia hospitalizations with lower chest wall indrawing.

For those sites at which radiography data were collected (Fiji, Gambia, Mongolia and Viet Nam), an experienced radiologist, blind to the clinical findings, reinterpreted all chest radiographs to determine the presence of primary end-point pneumonia according to WHO criteria.^14^^,^^15^

Where population data and a defined catchment population were available (Fiji, Gambia, Mongolia and Ho Chi Minh City in Viet Nam), annual incidence rates with 95% confidence intervals (CI) of hospitalizations from severe pneumonia according to both definitions, as well as the incidence of patients with primary end-point consolidation, were calculated per 100 000 infants aged 2–23 months. The differences in these incidence rates, with 95% CIs, were calculated using standard errors where appropriate. 

We conducted all statistical analyses using the software Stata, version 14.0 (StataCorp. LLC, College Station, United States of America).

## Results

Our analysis of the impact of the change in definition of severe pneumonia included 24 287 pneumonia hospitalizations of children aged 2–23 months (Table 1). The median ages of hospitalized pneumonia patients with severe pneumonia according to either the 2005 or 2013 definition ranged from 5 months in Hanoi to 12 months in Mongolia, and did not differ significantly by pneumonia case definition at each site (all *P* > 0.05; Table 2).

**Table 2:** Median age of pneumonia hospitalizations due to severe pneumonia in infants aged 2–23 months in six countries

WHO definition	Median age in months (interquartile range)^a^						
	Fiji	Gambia	Lao People's Democratic Republic	Malawi	Mongolia	Viet Nam	
						Hanoi	Ho Chi Minh City
2005^4^	9 (5 to 14)	10 (6 to 17)	11 (6 to 16)	8 (5 to 13)	12 (7 to 17)	5 (3 to 11)	9 (5 to 15)
2013^7^	8 (5 to 13)	11 (6 to 18)	11 (6 to 15)	8 (5 to 13)	12 (7 to 17)	5 (3 to 9)	9 (5 to 16)
*P*-value	0.346	0.126	0.789	0.156	0.275	0.924	0.640

WHO: World Health Organization.

^a^ Median ages of children hospitalized with severe pneumonia compared using the non-parametric *K*-sample test on the equality of medians.

The percentage of all-cause pneumonia hospitalizations with lower chest wall indrawing ranged from 64.5% (169/262) in Hanoi to 97.4% (15 300/15 709) in Malawi (Table 3). At six of the seven sites, the percentages of all-cause pneumonia hospitalizations due to severe pneumonia were significantly less (all *P* < 0.001) according to the 2013 WHO definition compared with the 2005 definition (Table 3). The percentage differences ranged from −7.1% (95% CI: −17.0 to 2.6) in Ho Chi Minh City to −49.9% (95% CI: −51.4 to −48.4%) in Malawi. When classifying severe pneumonia by the 2013 definition compared with the 2005 definition, the largest reductions were observed in data from the Gambia (−47.0; (733−285)/953) and Malawi (−49.9; (11 163−3327)/15 709). Between sites, the percentage of all-cause pneumonia hospitalizations due to severe pneumonia, according to the 2005 definition, varied from 50.0% (190/380) in Ho Chi Minh City to 81.9% (555/678) in Lao People's Democratic Republic; when adopting the 2013 definition, this ranged from 21.2% (3327/15 709) in Malawi to 56.6% (384/678) in Lao People's Democratic Republic. The percentage of severe pneumonia hospitalizations with primary end-point consolidation varied little within each site between the two definitions of severe pneumonia, from a reduction of 5.0% (1/71 to 10/155) at Hanoi to an increase of 1.2% in both the Gambia (60/285 to 146/733) and in Ho Chi Minh City (20/163 to 21/190). Between sites, however, the percentage of severe pneumonia hospitalizations with primary end-point consolidation varied from 6.5% (10/155) in Hanoi to 19.9% (146/733) in the Gambia according to the 2005 WHO definition, and varied from 1.4% (1/71) in Hanoi to 21.1% (60/285) in the Gambia using the 2013 definition.

**Table 3:** Properties of all-cause pneumonia hospitalizations in infants aged 2–23 months in six countries

Property of all-cause pneumonia	Fiji (*n* = 3254)	Gambia (*n* = 953)	Lao People's Democratic Republic (*n* = 678)	Malawi (*n* = 15709)	Mongolia (*n* = 3051)	Viet Nam	
						Hanoi (*n* = 262)	Ho Chi Minh City (*n* = 380)
**With lower chest wall indrawing**							
No. (%)	2665 (81.9)	711 (74.5)	443 (65.3)	15 300 (97.4)	2416 (79.2)	169 (64.5)	338 (88.9)
**With severe pneumonia**							
No. (%) according to the 2005 WHO definition^4^	2193 (67.4)	733 (76.9)	555 (81.9)	11 163 (71.1)	2337 (76.6)	155 (59.2)	190 (50.0)
No. (%) according to the 2013 WHO definition^7^	1838 (56.5)	285 (29.9)	384 (56.6)	3327 (21.2)	1422 (46.6)	71 (27.1)	163 (42.9)
Percent difference (95% CI)	−10.9 (−14.7 to −7.1)	−47.0 (−51.0 to −43.1)	−25.2 (−34.3 to −16.4)	−49.9 (−51.4 to −48.4)	−30.0 (−33.9 to −26.1)	−32.1 (−43.3 to −20.8)	−7.1 (−17.0 to 2.6)
**Of those with severe pneumonia, with primary end-point consolidation^a^**							
No. (%) according to the 2005 WHO definition^4^	238 (10.9)	146 (19.9)	NA	NA	268 (11.5)	10 (6.5)	21 (11.1)
No. (%) according to the 2013 WHO definition^7^	211 (11.5)	60 (21.1)	NA	NA	167 (11.7)	1 (1.4)	20 (12.3)
Percent difference (95% CI)	0.6 (−1.4 to 2.7)	1.2 (−2.7 to 5.8)	–	–	0.2 (−2.0 to 2.5)	−5.0 (−9.9 to −0.1)	1.2 (−5.9 to 8.4)

CI: confidence interval; NA: not available; WHO: World Health Organization.

^a^ Chest radiographs interpreted by independent radiologists, classifying primary end-point consolidation as per WHO definition.^14^

Table 4 summarizes the annual incidences of all-cause pneumonia hospitalizations due to severe pneumonia according to both 2005 and 2013 definitions of severe pneumonia, as well as the differences in these incidences. At all sites for which relevant data were available, the annual incidences were significantly less (all *P* < 0.001) according to the 2013 definition compared with the 2005 definition, ranging from a difference of −301.0 (95% CI: −405.2 to −196.8) in Fiji to −3242.6 (95% CI: −3695.2 to −2789.9) in the Gambia. Between sites, the annual incidence of pneumonia hospitalizations with primary end-point consolidation ranged from 160.7 (95% CI: 137.7 to 186.5) in Fiji to 1056.8 (95% CI: 892.3 to 1242.7) in the Gambia when severe pneumonia was classified according to the 2005 definition, and ranged from 138.4 (95% CI: 117.1 to 162.5) in Fiji to 529.9 (95% CI: 324.0 to 817.3) in Ho Chi Minh City when using the 2013 definition. The annual incidences of pneumonia hospitalizations with the presence of primary end-point consolidation were significantly different according to the definition of severe pneumonia used in data from Gambia, Mongolia and Ho Chi Minh City in Viet Nam (*P* < 0.001), but the difference was not significant in the data from Fiji (*P* = 0.09).

**Table 4:** Annual incidence of pneumonia hospitalizations with severe pneumonia in children aged 2–23 months in four countries

Incidence	Incidence per 100 000 infants^a^ (95% CI)			
	Fiji	Gambia	Mongolia	Viet Nam, Ho Chi Minh City
**All-cause pneumonia**				
Annual incidence according to the 2005 WHO definition^4^	1671.4 (1595.1 to 1750.5)	5305.4 (4928.3 to 5703.8)	4661.2 (4649.7 to 672.3)	5034.4 (4358.7 to 5780.9)
Annual incidence according to the 2013 WHO definition^7^	1370.4 (1301.3 to 1442.2)	2062.8 (1830.3 to 2316.8)	2836.2 (2824.7 to 2847.3)	4319.0 (3692.8 to 5017.1)
Difference	−301.0 (−405.2 to −196.8)	−3242.6 (−3695.2 to −2789.9)	−1825.0 (−2064.7 to −1585.3)	−715.4 (−718.5 to −712.3)
**With primary end-point consolidation^b^**				
Annual incidence according to the 2005 WHO definition^4^	160.7 (137.7 to 186.5)	1056.8 (892.3 to 1 242.7)	534.5 (515.1 to 552.9)	556.4 (344.8 to 849.3)
Annual incidence according to the 2013 WHO definition^7^	138.4 (117.1 to 162.5)	434.3 (331.4 to 559.0)	333.1 (314.1 to 351.9)	529.9 (324.0 to 817.3)
Difference	−22.3 (−55.0 to 10.4)	−622.5 (−826.1 to −418.8)	−201.4 (−283.0 to −119.9)	26.5 (25.4 to 27.5)

CI: confidence interval; NA: not available; WHO: World Health Organization.

^a^ Aged 2–23 months.

^b^ Chest radiographs interpreted by independent radiologists, classifying primary end-point consolidation as per WHO definition.^14^

Note: The table only presents data for sites with available population data and a defined catchment.

## Discussion

We have shown that, in six out of seven sites from the African and Western Pacific Regions, the percentage of pneumonia hospitalizations in children aged 2–23 months with severe pneumonia was significantly less using the 2013 definition of severe pneumonia compared with the 2005 definition. Similarly, at sites where incidence could be calculated, we have demonstrated that the annual incidence of pneumonia hospitalizations with severe pneumonia was significantly less using the 2013 definition of severe pneumonia compared with the 2005 definition. These findings are not unexpected given that the presence of lower chest wall indrawing (without danger signs) is no longer included in the 2013 WHO case definition for severe pneumonia;^8^ we calculated that 64.5 to 97.4% of hospitalized pneumonia cases had lower chest wall indrawing. 

An unpublished review (Nordgren M, GSK Vaccines, personal communication, 2016) of studies assessing the impact of the pneumococcal conjugate vaccine on pneumonia burden in infants younger than 2 years found much variability. These studies were observational or double-blinded randomized controlled trials, using the case definitions all-cause pneumonia, WHO-defined pneumonia (year not specified) and pneumococcal pneumonia. The varying sensitivity and specificity of the different case definitions^9^^,^^16^^,^^17^ mean that the magnitude of the impact of the vaccine on pneumonia calculated from studies of different design and using different case definitions cannot be directly compared.

Primary end-point consolidation has moderate sensitivity and specificity, and is the end-point which has shown reasonable consistency within clinical trials.^18^^,^^19^ However, in low- and middle-income countries, whether an infant receives a chest radiograph is often determined by clinical severity; those with danger signs (and not lower chest wall indrawing) are more likely to receive a radiograph compared with those without, irrespective of which WHO severe pneumonia definition is used. Unfortunately, we were unable to compare the percentage of pneumonia hospitalizations with danger signs, as each site used slightly different definitions of danger signs. This may explain why our findings show that, within each site, the changes to the definition of severe pneumonia had no significant effect on the percentage of severe pneumonia hospitalizations with radiological pneumonia. However, the incidence of pneumonia hospitalizations with primary end-point consolidation was significantly different, depending on the definition of severe pneumonia, within three of the four sites for which data were available.

Unsurprisingly, we observed variability in the incidence of pneumonia hospitalizations with severe pneumonia between sites, regardless of the case definition used. The incidence of hospitalized pneumonia is affected by many factors, including income, comorbidities and exposure to air pollution;^20^^,^^21^ variability between countries in hospitalized pneumonia burden is therefore to be expected. In addition, the incidence of pneumonia hospitalizations with severe pneumonia is also influenced by access to care and admission criteria.^22^ For example, despite being tertiary facilities, both Vietnamese sites had a lower percentage of pneumonia admissions that were severe according to the 2005 definition than compared with other sites, indicating a lower threshold for admission and/or better health-seeking behaviour. A recent study has reported that many children with non-severe respiratory disease are admitted to primary, secondary and tertiary care facilities in Viet Nam.^23^ We found that the presence of lower chest wall indrawing varied between the two Vietnamese sites, suggesting that the admission criteria also vary between these hospitals. Furthermore, the diagnosis of lower chest wall indrawing is observer dependent, also affecting within-site observations.^24^ Although the presence of danger signs was not recorded consistently between the sites, the key clinical criteria of severe pneumonia using either the 2005 or 2013 severe pneumonia definition (age-specific respiratory rate, cough and presence of lower chest wall indrawing) were recorded consistently at each site, making within-site comparisons possible. 

The change from the 2005 to 2013 severe pneumonia case definition aimed to simplify the pneumonia treatment algorithm for community health workers and reduce unnecessary burden on families and health-care services.^25^ However, our findings need to be considered when discussing changes in the epidemiological impact on pneumonia burden and pneumonia impact evaluation. If clinicians have changed their admission criteria for pneumonia in accordance with changes in WHO guidelines, then the incidence of pneumonia hospitalizations will appear to decline regardless of any intervention, because of children with pneumonia and lower chest indrawing (no danger signs) now being managed as outpatients. For pneumonia epidemiology and pneumonia intervention evaluations, our findings highlight the importance of stating which WHO definition is used and whether admission criteria changed during the observation period. Further studies are also required to understand the impact of this definitional change in high-mortality settings, where rates of bacterial pneumonia are increased and lower chest wall indrawing has been identified as an independent risk factor for mortality.^26^
